# Diagnosis of spontaneous bacterial peritonitis in infants and children with chronic liver disease: A cohort study

**DOI:** 10.1186/1824-7288-37-26

**Published:** 2011-05-21

**Authors:** Mortada HF El-Shabrawi, Ola El-Sisi, Sawsan Okasha, Mona Isa, Sayed Abou Elmakarem, Iman Eyada, Zainab Abdel-Latif, Gamal El-Batran, Naglaa Kamal

**Affiliations:** 1 Pediatric Department, Faculty of Medicine, Cairo University, Cairo, Postcode 12411, Egypt; 2 Chemical Pathology Department, Faculty of Medicine, Cairo University, Cairo, Postcode 12411, Egypt

## Abstract

**Background:**

Spontaneous bacterial peritonitis (SBP) is a serious complication in infants and children with chronic liver disease (CLD); however its diagnosis might be difficult. We aimed to study the feasibility of diagnosing SBP by routine ascitic fluid tapping in infants and children with CLD.

**Methods:**

We enrolled thirty infants and children with biopsy-proven CLD and ascites. Ascitic fluid was examined for biochemical indices, cytology and cell count. Aerobic and anaerobic bacteriological cultures of ascitic fluid were preformed. Direct smears were prepared from ascitic fluid deposit for Gram and Zheil-Nelson staining.

**Results:**

Patients were divided into three groups: Group I included five patients with SBP in which the cell count was ≥ 250/mm^3 ^and culture was positive (16.7%), Group II, eight patients with culture negative neutrocytic ascites (CNNA) with cells ≥ 250/mm^3 ^and negative culture (26.7%) and Group III, seventeen negative patients (56.6%) in which cells were <250/mm^3 ^and culture was negative. None of our patients had bacteriascites (i.e. culture positive with cells <250/mm^3^). Presence of fever was significantly higher in SBP and CNNA. The mean lactate dehydrogenase (LDH) level was significantly higher in ascitic fluid in the infected versus sterile cases (*p *< 0.002). A ratio of ascitic/serum LDH ≥ 0.5 gave a sensitivity of 80%, specificity of 88%, positive predictive value (PPV) of 66.7%, negative predictive value (NPV) of 93.7% and accuracy of 63.3%. The mean pH gradient (arterial - ascitic) was significantly higher in SBP and CNNA cases when compared to the negative cases (p < 0.001). Ascitic fluid protein level of ≤ 1 gm/dl was found in 13/30 (43.3%) of studied cases with a sensitivity of 100%, specificity of 64.7%, PPV of 45.5%, NPV of 100% and diagnostic accuracy of 53.3% (p = 0.0001).

**Conclusions:**

SBP is a rather common complication in children with CLD. Culture of the ascitic fluid is not always diagnostic of infection. Biochemical parameters of the ascitic fluid definitely add to the diagnostic accuracy. LDH ascitic/serum ratio ≥ 0.5, an arterial-ascitic pH gradient ≥ 0.1 and total ascitic fluid protein ≤ 1 gm/dl are the most significant parameters suggesting infection.

## Background

Patients with chronic liver disease (CLD) are particularly susceptible to infections with a higher prevalence in cirrhotics [1]. Ascites is a frequent complication of cirrhosis. Spontaneous bacterial peritonitis (SBP) is defined as infected ascites in absence of recognizable secondary cause of infection [2]. It occurs in up to 10% of adult CLD patients with ascites because of bacterial overgrowth with translocation through a more permeable small intestinal wall and impaired defense mechanism [3]. SBP can have a silent up to catastrophic presentation. However, patients typically have nonspecific symptoms such as nausea, abdominal pain, fever or mild confusion [4]. Fever and abdominal pain are among the most common presentations [5]. SBP is one of the major causes of morbidity and mortality in cirrhotic patients [1]. Lethality is high. Older studies reported 80-100% lethality in SBP, which is probably given partly by the worse therapeutic possibilities in cirrhotic patients and lack of availability of effective antibiotics. Better results with only 20%-40% lethality reported in more recent studies are, to a certain extent, due to early diagnosis and treatment [6-8].

The aim of this study was to prospectively evaluate the feasibility of diagnosing SBP by routine ascitic fluid tapping in a cohort of Egyptian infants and children with biopsy-proven CLD associated with ascites in order to study the prevalence of SBP in them, its different clinical presentations, causative organisms, diagnosis and prognosis. We also aimed to study the value of biochemical parameters including ascitic fluid pH, lactate dehydrogenase (LDH), glucose and total proteins compared to their blood levels in diagnosing culture-negative patients.

## Patients and methods

We prospectively enrolled thirty hospitalized infants and children from the Pediatric Hepatology Unit of Cairo University Children Hospital with CLD confirmed by liver biopsy and associated with ascites.

All patients were subjected to the following:

1) Full history-taking

2) Thorough clinical examination

3) Routine laboratory investigations including: complete blood count, biochemical tests of liver function, total plasma proteins and serum albumin, and kidney function tests.

4) Serum complement (C) C_3_, C_4 _and immunoglobulins (Igs) M, G and A (using Radial Immune Diffusion).

5) Diagnostic paracentesis was performed under strict aseptic conditions and the amount of fluid withdrawn for the diagnosis did not exceed 40 ml. Therapeutic large volume paracentesis followed immediately when indicated.

Ascitic fluid was examined for:

I. Biochemical indices, including pH, glucose, LDH and total proteins.

II. Cytology and cell count.

III. Bacteriological examination. A portion of ascitic fluid (20 ml) was allocated into two test tubes containing thioglycollate broth medium, which were incubated at 37°C. The remainder of ascitic fluid was immediately centrifuged and the deposit was inoculated on trypcase soy blood agar for aerobic and anaerobic cultures and on to MacConkey's and Thio sulfate Citrate Bile Salts Sucrose (TCBS) agar medium for aerobic culture. Plates were examined after 24 hours (hr) and again after 48 hr. If they showed no growth, subcultures were done from thioglycollate broth and nutrient broth and on to blood agar and Mac Conkey's medium for aerobic cultivation; broth media were incubated up to 10 days. Anaerobic colonies were identified by API-20 A kit. Direct smears were prepared from ascitic fluid deposit for Zheil-Nelson (ZN) and Gram staining. Following cytological and bacteriological examination, patients were classified into 3 groups according to the long-accepted classification [6,7,9]:-

**Group I **spontaneous bacterial peritonitis (SBP) in which the cell count was ≥ 250/mm^3 ^and culture was positive (five cases or 16.7%).

**Group II **culture negative neutrocytic ascites (CNNA) with cells ≥ 250/mm^3 ^and culture negative (eight cases or 26.7%).

**Group III **negative cases (seventeen cases or 56.6%) in which cells ≤ 250/mm^3 ^and culture negative.

**6) **Arterial blood samples were taken for pH determination by Bayer Ciba-Corning 288 Blood Gas Analyzer

7) Blood and ascitic fluid were examined for the following biochemical parameters:

A) pH.

B) LDH was measured by ultraviolet kinetic method based on the following equation:

C) Glucose was measured by an Express Plus automated random access autoanalyzer, using an enzymatic method depending on the following equation:

D) Total proteins and albumin were measured by automated auto-analyzer 550 express plus (Ciba Corning Diagnostics).

All procedures were in accordance with the current revision of the Helsinki Declaration [10]. Parents or guardians of all patients had to give informed consents to participate in the study. Patients/guardians who refused to consent were excluded.

## Statistical Analysis

The power of work of the study was more than 90%. All results were statistically analyzed. Qualitative variables, expressed as percentages, were compared in different groups using the Chi-square test. For all the statistical tests done, a p-value < 0.05 indicated a significant result. Diagnostic accuracy of different biochemical tests were measured by multi-variate analysis.

## Results

This study included thirty infants and children with CLD confirmed by liver biopsy and associated with ascites. Their ages ranged from 6 months to 11 years, with a mean age of 5.1 ± 3.3 years. There were nineteen males (63.4%) and eleven females (36.6%). The study groups were classified into: Group I, five patients with SBP (16.7%), Group II, eight patients with CNNA (26.7%) and Group III, seventeen negative patients (56.6%). None of our patients had bacteriascites, i.e., culture positive with cells < 250/mm^3^. The etiology of CLD in study group is summarized in Table 1.

**Table 1:** Classification of study groups according to etiology of CLD

Etiology of CLD	SBP cases N = 5 (N)	CNNA cases N = 8 (N)	Negative cases N = 17 (N)	Total N = 30 N(%)
Veno-occlusive disease (VOD)	1	4	9	14 (46.6%)

Liver cirrhosis	3	1	5	9 (30%)

Neonatal giant cell hepatitis	-	-	2	2 (6.6%)

Hepatic fascioliasis	-	1	1	2 (6.6%)

Portal vein obstruction	1	-	-	1 (3.4%)

Cryptogenic liver cirrhosis	-	1	-	1 (3.4%)

Caroli's Disease	-	1	-	1 (3.4%)

There were no statistically significant differences in the clinical characteristics between the three studied groups with the exception of fever which was more evident in patients with SBP (100%) and CNNA (87.5%) when compared to negative cases (53%) {p < 0.05}. Oral broad spectrum antibiotics (mostly first generation cephalosporins or amoxicillin-clavulanate were given for 10 days) were given in 4/8 (50%) of those with CNNA before presentation. None of the positive or negative cases received oral antibiotics in the immediate pre-presentation time.

The standard biochemical liver function tests, total plasma proteins and serum albumin and kidney function tests were comparable between the study groups. The mean serum IgG, IgM, and IgA level did not differ between the three groups. The CNNA group exhibited significantly higher levels of serum IgM and IgG when compared to negative cases (p < 0.05); this was not observed in the SBP group. C_3 _level was highest in CNNA (118.5 ± 51.5) when compared to negative cases (78.8 ± 29.4) (p < 0.05), but C_4 _did not significantly differ in the three groups. Leucocytosis with shift to the left, as an indicator of infection, was observed in 20% of SBP group, in 29.4% of negative cases, and in 50% of those with CNNA.

In this study, positive culture was detected in five cases (SBP), two of them reveled *Bacteroides*, gram negative anaerobes (40%), coagulase positive *Staphylococcus *in one case (20%), anaerobic *Streptococcus* in one case (20%) and one case had *Streptococcus pnemoniae *(20%).

The mean cell count in ascitic fluid was significantly higher (p < 0.01) in SBP (906 ± 1178.8) and CNNA ( 1013.7 ±1322.5) when compared to negative cases (60.6 ± 72.8). Mean pH of the ascitic fluid was 7.37 ± 0.03 in SBP cases, 7.36 ± 0.05 in CNNA, which were significantly lower than 7.43 ± 0.05 in negative cases (p < 0.05).

**Figure 1:**
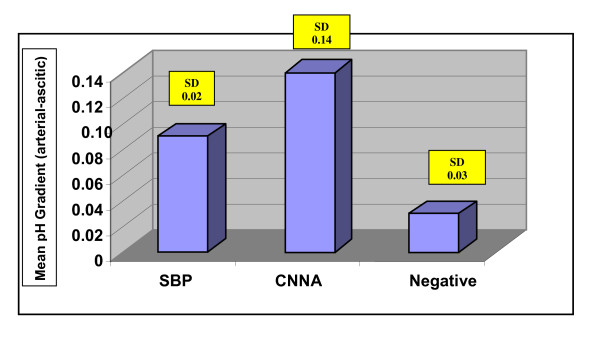
**Histogram for the mean values (±SD) of pH gradient (Serum-asitic fluid) in different studied groups**.

The mean pH gradient (arterial - ascitic) was significantly higher in SBP and CNNA cases when compared to the negative cases (p < 0.001) (Figure 1). A pH gradient at a cut off ≥ 0.1 (Table 2) was found to have a sensitivity of 80%, specificity of 94%, PPV of 80%, NPV of 94.1% and diagnostic accuracy of 66.7% (p = 0.0001).

**Table 2:** The values of different biochemical parameters in studied groups

Groups	pH gradient (arterial - ascitic)		Glucose level in ascitic fluid (mg/dl)		Glucose gradient (serum-ascitic fluid)		LDH ratio ascitic fluid/serum		Total proteins level of ascitic fluid	
	
	≥ 0.1	<0.01	≤60	>60	≥ 60	<60	≥ 0.5	<0.5	≤1 gm%	>1 gm%
**SBP** **(n = 5)**	4/5 (80%)	1/5 (20%)	3/5 (60%)	2/5 (40%)	3/5 (60%)	2/5 (40%)	4/5 (80%)	1/5 (20%)	5/5 (100%)	-

**CNNA** **(n = 8)**	6/8 (75%)	2/8 (25%)	6/8 (75%)	2/8 (25%)	5/8 (62.5%)	3/8 (37.5%)	5/8 (62.5%)	3/8 (37.5%)	2/8 (25%)	6/8 (75%)

**Negative** **(n = 17)**	1/17 (6%)	16/17 (94%)	3/17 (17.6%)	14/17 (82.4%)	2/17 (11.7%)	15/17 (88.3%)	2/17 (11.7%)	15/17 (88.3%)	6/17 (35.2%)	11/17 (64.7%)

**P value**										

**SBP versus negative**	p < 0.001		p > 0.05		p < 0.05		p < 0.002		p < 0.05	

**SBP versus CNNA**	p > 0.05		p > 0.05		p > 0.05		P > 0.05		p < 0.05	

**CNNA versus negative**	p < 0.001		p < 0.05		p < 0.05		p < 0.01		p > 0.05	

**Figure 2:**
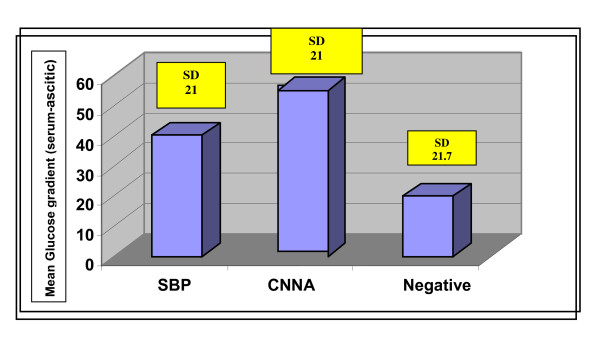
**Histogram for the mean values (±SD) of glucose gradient mg/dl (Serum-asitic fluid) in different studied groups**.

Regarding the glucose level in the ascitic fluid, the mean level was 75 ± 35.7 mg/dl, 56.8 ± 17.7 and 86.3 ± 22.3 in SBP, CNNA and negative cases respectively. The mean gradient of glucose (serum-ascitic) is presented in Figure 2. CNNA cases had significantly lower levels and higher gradient if compared to negative cases (p < 0.05); this was not observed in SBP cases. A cut off value for serum glucose level ≤ 60 mg/dl, (Table 2) had a total sensitivity of 60%, specificity of 82%, PPV of 50%, NPV of 87.5%, and diagnostic accuracy of 56.7% (p = 0.0001). A glucose gradient of ≥ 60 mg/dl (Table 2), had a sensitivity of 60% and specificity of 88%, PPV of 60% NPV of 88.2% and diagnostic accuracy of 60% (p = 0.0001).

LDH level in ascitic fluid had a mean of 132 ± 50.7 and 110.8 ± 47.3 in SBP and CNNA respectively, which was significantly high if compared to 61.5 ± 33.6 in negative cases (p < 0.002). The ratio of LDH (ascitic/serum) was 0.5 ± 0.08 and 0.5 ± 0.16 in SBP and CNNA respectively, which is significantly higher than 0.3 ± 0.18 in negative cases (p < 0.05). An LDH ratio at cut off ≥ 0.5 (Table 2) had a sensitivity of 80%, specificity of 88%, PPV of 66.7%, NPV 98.75% and diagnostic accuracy of 63.3% (p = 0.0001).

Total ascitic fluid protein level was lowest in SBP (0.6 ± 0.4 gm/dl), 2.9 ± 2 gm/dl in CNNA and 1.7 ± 0.09 in negative cases (p < 0.05). A protein level of ≤ 1 gm/dl was found in 13/30 (43.3%) of studied cases (Table 2) with a sensitivity of 100%, specificity of 64.7%, PPV of 45.5%, NPV of 100% and diagnostic accuracy of 53.3% (p = 0.0001). Regarding the outcome, five patients died during the enrollment period, giving a mortality of 33.3%, two of them had SBP diagnosed as liver cirrhosis, one had CNNA diagnosed as Caroli's disease and two were negative cases (diagnosed as Neonatal giant cell hepatitis and liver cirrhosis).

## Discussion

In this study, thirty children with CLD and ascites were studied for evidence of peritonitis, five (16.7%) of them diagnosed as SBP and 8 (26.7%) had CNNA, i.e., total cases with evidence of peritonitis were 43.4%.

The incidence of peritonitis whether SBP or CNNA is relatively high in this study if compared with Syed et al, who reported an incidence of 20/81 (24.6%) peritonitis in cases with CLD. Classical SBP was noted in four cases, CNNA was present in thirteen cases and bacterascites was found in three of their patients [2]. Meanwhile, Dehghani et al., reported an incidence of SBP in 36.1% children with chronic end-stage liver disease [11]. The prevalence of SBP was 20.6% in a similar study conducted by Haghighat and colleagues [5]. A lower prevalence for SBP was noted in a retrospective study performed by Evans and colleagues. The prevalence for SBP and CNNA was 1.4% and 2.1% respectively (giving a combined prevalence of 3.9%) [12]. On the other hand, none of the cirrhotic cases with chronic ascites in the study conducted by Romney et al., showed evidence of SBP [13]. In concordance, Jeffries et al. reported that the prevalence of SBP in patients with CLD was null, but the prevalence of bacterascites was 2.3% [14].

Clinical signs and symptoms such as fever and abdominal pain are considered the most prevalent features of SBP [15]. In the present study, fever was the only significantly evident clinical feature in the infected (SBP + CNNA), (92.3%) when compared with negative cases (53%) (p < 0.05). Similarly, Vieira and colleagues found that fever, worsening ascites and encephalopathy were the most prevalent clinical features in their patients with infected ascites but the difference between infected and non-infected cases was not significant [16]. Another study reported that upper gastrointestinal bleeding and abdominal pain were the most common presenting symptoms of SBP [2].

Song et al. and Christou et al. reported that bacteria of enteric origin, especially *Escherichia coli *and *Klebsiella *species were the most common pathogens encountered in infected cases [1,17]. Similarly, a positive culture with enteric bacteria was observed in 60% (3/5) of our SBP group.

The biochemical liver function tests, serum albumin and total proteins did not differ significantly between the three groups. Natarajan et al. and Wojtacha et al., reported a high incidence of bilirubin, liver enzymes, albumin, total protein disturbance in patients with SBP when compared to the non-infected cases [18,19]. Liver dysfunction is known to impair the defense mechanisms against infection seen in cirrhotic patients because of depressed reticuloendothelial system phagocytic activity, reduced serum complement levels and low antibacterial activity of asitic fluid [18].

The most important predictor of renal failure and hospital mortality after SBP is the presence of renal impairment at infection diagnosis [20]. Serum urea and creatinine did not differ significantly in our study in the various groups. Vieira and colleagues also reported no significant difference in serum urea and creatinine between infected and sterile ascites cases [16].

The immunological status of patients, demonstrated by the serum Igs (IgM, IgG, IgA), C_3 _and C_4 _levels did not differ significantly in SBP cases when compared to CNNA or negative cases. Yildirim et al., reported decreased levels of serum and ascitic C_3 _and C_4 _in patients with SBP when compared to those without, but the serum Igs were similar in the patients with and without SBP [21]. The complement components have been shown to play an important role in the development of immunologically mediated inflammatory reactions. Cirrhotic patients are known to have low ascitic fluid C_3 _and are more predisposed to SBP [21]. Therefore C_3 _and C_4 _levels must be determined in the ascitic fluid itself as the possibility of alternative complement pathway activity occurring within the peritoneal cavity cannot be ruled out.

The pH of ascitic fluid was significantly lower in both SBP and CNNA when compared to negative cases. On the other hand, Vieira et al., reported a median pH level of 7.4 in the non-infected as well as in the infected ascites group [16]. The ascitic fluid pH appears to be an indirect measurement of the presence of PMNL and large numbers of PMNL must be present before the pH decreases.

In this study, an ascitic fluid pH lower than 7.35 and a blood-ascitic fluid pH gradient of 0.1 or greater were the most accurate thresholds for diagnosing SBP as evidenced by having the highest diagnostic odds ratio. Conversely, a blood-ascitic fluid pH gradient of lower than 0.1 lowers the likelihood of SBP [4].

The ascities median glucose level was lower in infected cases (88.5 mg/dl) when compared to the non-infected cases (104 mg/dl) in a study conducted by Vieira and colleagues, but this difference was not statistically significant (p = 0.07) [16]. Yilidrim et al., reported a mean serum glucose level of 96 mg/dl in cases with SBP when compared to 85.4 mg/dl and 70.5 mg/dl in malignant and tuberculous ascities respectively [21]. In contrast to cerebrospinal fluid infection, the glucose levels or the serum-ascites glucose ratio are not useful to diagnose SBP. This may be explained by the low concentration of bacteria frequently observed in cases of SBP in contrast with observations in secondary bacterial peritonitis [15].

The mean LDH level in this study was significantly higher in the infected versus sterile cases (p < 0.002). No significant differences in LDH level between infected and non-infected cases of ascites was reported in Vieira study [16].

The total proteins estimated in ascitic fluid were significantly lower in cases with SBP when related to the CNNA or negative cases. Such et al., reported that patients with SBP, had a significantly decreased serum albumin and ascitic fluid total proteins. Total proteins ≤ 1 gm/dl was found to be the most important risk factor for development of SBP in cirrhotic patients [22]. It is related to the low ascitic fluid bactericidal capacity observed in these patients [23].

Mortality rate observed in this study was 16.7% (5/30) and it did not differ significantly in the three groups 40%, 12.5%, 12%, respectively (p > 0.05). In reports from the 1970s, the mortality rate from SBP exceeded 90% but recent data show a lower mortality rate of 30% [24,25].

## Conclusion

We conclude that SBP is a rather common problem in children with CLD. Apart from fever, symptoms and signs are not always conclusive of the diagnosis and a high index of suspicion is needed in all cases with ascites for early diagnosis of cases. Routine culture of the ascitic fluid is not always diagnostic of infection. Biochemical parameters of the ascitic fluid will add to the diagnostic accuracy. A pH gradient (arterial-ascitic) ≥ 0.1, LDH ratio ascitic/serum ≥ 0.5 and total proteins of ascitic fluid of ≤ 1 gm/dl are the most important parameters highly suggestive of the presence of infection.

## Abbreviations

(CLD): Chronic liver disease; (C): Complement; (CNNA): Culture negative neurocytic ascites; (Igs): Immunoglobulins; (LDH): Lactate dehydrogenase; (NPV): Negative predictive value; (PPV): Positive predictive value; (SBP): Spontaneous bacterial peritonitis; (TCBS): Thio sulfate Citrate Bile Salts Sucrose and (ZN): Zheil-Nelson.

## Competing interests

The authors declare that they have no competing interests.

## Authors' contributions

All authors read and approved the final manuscript.

MS and ZA contributed equally to this work;

MS, SO and ZA recruited the patients;

MS, SO, IE and SA clinically supervised the patients;

OS contributed reagents/analytic tools;

MI analyzed the data;

MS and MI wrote up the manuscript.
